# Tumours of the Groin and Their Differential Diagnosis

**Published:** 1896-07-04

**Authors:** W. Ernest Miles

**Affiliations:** House Surgeon to St. Mark's Hospital for Diseases of the Rectum; late House Surgeon to the Metropolitan Hospital, and to the Radcliffe Infirmary, Oxford


### TUMOURS OF THE GROIN AND THEIR DIFFERENTIAL DIAGNOSIS.

By W. Ernest Miles, F.R.C.S.Eng., House Surgeon to St. Mark's Hospital for Diseases of the Rectum; late House Surgeon to the Metropolitan Hospital, and to the Radcliffe Infirmary, Oxford.

Tumours of the groin are both so frequent and variable in kind and origin, while their differential diagnosis is often so obscure and puzzling, that I have thought that a consideration of them collectively with a view to comparing and contrasting their especial signs and symptoms may be useful to practitioners. For the sake of convenience in description, I propose to consider these tumours in two divisions, namely (1) those arising from the superficial structures, that is, from the skin and superficial fat and fascia; (2) those originating in or brought into relation with the deeper tissues, that is to say, those structures situated beneath the deep fascia.

**I. Tumours Arising from Superficial Structures.—** (A) From the skin.

(a) *Sebaceous cysts* are occasionally met with in the groin, and vary in size from that of a pea to that of an orange. They are smooth, round or oval, movable under the integument, sometimes solid to the touch, though occasionally elastic or semi-fluctuating. Usually a small dark spot can be detected on the surface, which is the duct of the sebaceous gland blocked by dirt or epithelial *débris*. When the skin is pinched up from the surface of the tumour it is found to be adherent at this spot only. A sebaceous cyst will have to be distinguished from a lipoma, a chronic abscess, and a gumma. A lipoma, though sometimes smooth, is more commonly lobulated; the skin is freely movable over its surface, but when pinched up between the fingers a peculiar dimpling will be noticed, indicating its attachment by fibrous septa to the capsule of the fatty growth. Moreover, if the margin of the growth is pressed upon by the finger, a lipoma will slip away in a characteristic manner. A chronic abscess is less tense to the touch, softer, cannot be moved under the skin, and is adherent to the tissues immediately surrounding it; fluctuation can also be obtained. A gumma is adherent to the skin and underlying structures, decidely infiltrates the surrounding tissues, is of recent growth, and is especially liable to soften at one or more places.

(b) *Papillomata*, or warty growths, are sometimes met with in the groin. They consist of outgrowths from the skin entirely raised above its surface, and definitely marked off at the attached base. They are dry, firm, and hard, and are usually branched on the surface, resembling somewhat the appearance of a cauliflower. The skin in the immediate vicinity of the attached base is soft and pliable, showing that there is no infiltration of the surrounding tissues by the growths. If such infiltration does occur the papilloma has become carcinomatous, and must then be considered malignant. Induration and enlargement of the nearest lymphatic glands will confirm this view.

(c) *Moles.*—These are pigmented patches, varying in size and always congenital. They are often raised above the surface of the surrounding skin, and not unfrequently covered with hair. As a rule they remain stationary throughout, but occasionally increase in size; become more prominent and resemble papillomata in structure (pigmented wart). They are also prone to become malignant, and are the starting point of a growth of sarcomatous nature known as melanotic sarcoma.

(d) *Melanotic sarcomata* occur as darkly pigmented prominent masses, soft to the touch and of rapid growth. They are usually smooth on the surface, but not uncommonly become ulcerated early and bleed readily. They are exceedingly malignant and soon implicate the nearest lymphatic glands. Secondary deposits in the viscera take place rapidly, and it is said that when the growth has attained the size of a filbert dissemination of the disease has already began. The diagnosis of the malignant nature of these growths may be based upon the speedy enlargement of pre-existing moles, the early implication of lymphatic glands, and especially by the appearance of pigmented patches in the skin immediately surrounding them. If, of course, the liver is found enlarged and patches of dulness discovered in the lungs, the diagnosis is substantiated. Melanotic sarcomata have to be distinguished from pigmented warts which are not malignant, are of slow growth, firm to the touch, and either lobulated or pedunculated. It must be borne in mind that pigmented warts may themselves become carcinomatous, when their increased rate of growth may afford reason for suspecting sarcomatous change, but their hard, indurated character, and especially the induration of the adjacent skin, together with the more speedy implication of the glands, will suffice to distinguish their true nature. Nevertheless, if any pigmented mole, after having been stationary, suddenly shows signs of increasing in size, it must be regarded with suspicion.

(e) *Sebaceous adenomata*, arising from the sebaceous follicles, have been occasionally met with, but are very rare. They form lobulated masses, definitely raised above the surrounding skin, and may attain great size. They are of slow growth, and after existing for some time may ulcerate and so resemble squamous carcinoma. The absence of infiltration of the surrounding tissues, together with the absence of glandular implication, will serve to distinguish this form of tumour from carcinoma. The only other condition that a sebaceous adenoma may be confounded with is tuberculous deposit in the skin. In the latter condition, the general constitutional condition of the patient, the probable multiplicity of the lesions, the tendency of these growths to soften and break down, and if ulceration has supervened, the undermined irregular character of the margins of the ulcer, together with the flabby, unhealthy appearance of the granulations, will serve to emphasise their tuberculous nature.

(*f*) *Keloid.*—Growths of this nature may attack scars in the groin as in other situations, and are then known as cicatricial keloid. Occasionally they arise spontaneously, whence the term spontaneous keloid. They are essentially new growths of the connective tissue type, characterised by one or more irregularly-shaped, variously-sized, elevated, smooth, firm, somewhat elastic, reddish cicatriform lesions. The reddish colouration is best seen while the growth is still increasing in size, and is due to the numerous blood vessels which it contains. Later on these vessels may become obliterated in great measure, imparting to the growth a peculiar wax-like appearance. The signs of keloid are so striking in character that it would be difficult to confound it with anything else. It is, however, most likely to be mistaken for an exuberant cicatrix, from which it may be distinguished by its colour, outline, elevation, and consistence, and frequently by the presence of pain.

(*g*) *Squamous carcinoma* may attack warts or scars in this region. The growth is usually circumscribed, hard and elevated. It rapidly infiltrates the surrounding tissues whereby it becomes fixed and the skin in its near vicinity is indurated. The neighbouring lymphatic glands will most commonly be found enlarged. The growth steadily increases in size, and is very prone to ulcerate on the surface. When this occurs the margins of the ulcer are sinuous, everted, and indurated, and the floor of the ulcer looks unhealthy and devoid of granulations. This form of growth has to be distinguished from a sebaceous adenoma and a simple warty excrescence. The fact that the growth infiltrates surrounding structures soon affects neighbouring lymphatic glands, grows rapidly, has a distinct tendency to ulcerate, will render the distinction from either of these an easy matter.

**B. Tumours Originating in the Subcutaneous Structures.**—The subcutaneous tissue consists of fat and connective tissue, contained in which are blood vessels, nerves, lymphatic glands, and lymphatic vessels. Since each of these structures may give rise to tumours we shall consider them separately.

(*a*) From fatty tissue—(1) lipomata; (2) nævo-lipomata; (3) myxo-lipomata.

(1) *Lipomata* occur as circumscribed, soft, pseudo-fluctuant, inelastic growths. They grow slowly, and may attain a large size, in which case they are liable to become ulcerated on the surface from continued friction against the clothes. They are also prone to shift their position under the influence of gravity, showing a migratory tendency which is very characteristic. The diagnosis is simple, and is confirmed by observing the dimpling of the skin when pinched up from its surface, and also by the tumour slipping away when pressed upon at its edge.

(2) *Nævo-lipomata* resemble closely pure lipomata, but differ in this respect, that they alter in size according as the patient is in the upright or recumbent position. If, then, a tumour manifesting the signs of a lipoma should diminish in size when the patient is lying down, and more especially if it should become smaller still when compressed by the hand, the inference may safely be drawn that the tumour in question is a nævo-lipoma.

(3) *Myxo-lipomata* cannot be distinguished clinically with any degree of certainty from ordinary lipomata.

(*b*) From connective tissue (1) soft fibromata; (2) molluscum fibrosum; (3) gummata; (4) congenital cystic hygromata.

(1) *Soft fibromata*, or fibro-cellular tumours, are usually circumscribed, soft, smooth, globular, painless tumours, but occasionally they are pedunculated, and may then attain a large size. When these tumours are pedunculated and exist singly they are easily recognised. If they exist in numbers, the disease is in all probability molluscum fibrosum. When not pedunculated it is difficult to distinguish them from lipomata or myxomata.

(2) *Molluscum fibrosum.*—This is a connective tissue new growth, characterised by sessile or pedunculated, soft or firm, rounded, painless tumours, varying in size from a pea to an egg or larger, situated beneath and in the skin. These tumours are multiple, existing in hundreds in the same patient. The diagnosis is easy.

(3) *Gummata* are sometimes situated in the connective tissue. They are of recent growth, of ill-defined outline, immovable in the surrounding tissues, and having a tendency to infiltrate surrounding structures, such as skin, fascia, and muscles. They are prone to soften at one or more points, and may slough, the resulting ulcer having steep punched-out margins and a floor resembling a piece of wash-leather. The diagnosis will have to be made from a sebaceous cyst, a chronic abscess, and a lipoma. A history of syphilis, with perhaps co-existing syphilitic lesions in other parts of the body; the absence of defined outline, and especially the infiltrating mode of growth, will render the diagnosis certain.

(4) *Congenital cystic hygromata.*—These tumours are always congenital, and are sometimes met with in the groin, though their more common seat is the neck or axilla. They may exist singly or otherwise—in the latter case the others will in all probability be found in the above-mentioned situations. They are irregular in outline, very soft and loose, and of unequal consistency, fluctuating in some parts and soft solid in others. The skin over the tumour is especially liable to repeated attacks of inflammation, which feature is of great diagnostic value. The fact that these tumours are congenital serve to distinguish them from any other.

(*c*) From the subcutaneous vessels—(1) nævus; (2) varix; (3) hæmatoma.

(1) *Nævi*, when met with in the groin, as in other situations, are congenital, or appear soon after birth. It is the venous nævus that engages our attention here, the capillary variety being merely a discolouration of the skin rather than a definite tumour, when considered from a clinical point of view. These tumours may be stationary or grow with varying degrees of rapidity. Although they consist chiefly of fluid, fluctuation cannot be obtained, since the fluid is contained in numerous small spaces (dilated veins) rather than in a single cavity, where the fluctuation wave has room for development. Since the blood vessels consist chiefly of veins, these tumours do not pulsate. Under compression they are to some degree reducible, but the reduction takes place gradually, and not with the sudden slip or gurgle so characteristic of hernia. The diagnosis is simple as a rule. If a capillary nævus coexists in the skin over the tumour, and if the growth is of a bluish colour and becomes fuller and more tense during an expiratory effort, a subcutaneous nævus certainly exists.

(2) *Varix.*—Varicose veins are commonly met with in this locality, the vein that is most usually affected being the internal saphenous.

(3) *Hæmatomata.*—These swellings are met with as a result of injury, and are due to extravasation of blood from torn subcutaneous vessels. They are of variable size and generally circumscribed. When recent they are soft and fluctuating, but may be hard and solid-feeling when of longer standing, owing to coagulation of the extravasated blood. The history of an injury, such as a blow or a puncture with a sharp instrument, the suddenness of the appearance of the swelling, and the presence of discolouration of the skin over and around it, will enable the diagnosis to be made. It must be borne in mind that a hæmatoma frequently suppurates and then presents the sign of an acute abscess. If a swelling that has appeared suddenly after the receipt of an injury, and which has remained stationary for forty-eight hours or so, then progressively grows larger, is surrounded by œdema, and there is great heat and redness in the part accompanied by severe pain of a throbbing character, together with a rapid rise of temperature, with or without an initial rigor, we have to deal with a suppurating hæmatoma.

(*d*) From the lymphatic glands.

We may recall the well-known fact that the lymphatic glands of the groin are arranged in two sets, and receive lymph from distinct and separate regions. The horizontal set is arranged along and parallel to Poupart's ligament, and is known as the inguinal group, receiving lymph from the lower part of the abdomen, the penis and scrotum in the male and the vulva in the female, the perineum and the buttock. The vertical set is clustered round the saphenous vein at the point where it penetrates the cribriform fascia of the saphenous opening. These glands are called femoral, and receive lymph from the whole of the lower extremity.

